# High fives motivate: the effects of gestural and ambiguous verbal praise on motivation

**DOI:** 10.3389/fpsyg.2014.00928

**Published:** 2014-08-27

**Authors:** Bradley J. Morris, Shannon R. Zentall

**Affiliations:** ^1^Department of Educational Psychology, Kent State UniversityKent, OH, USA; ^2^School of Family and Consumer Sciences, University of AkronAkron, OH, USA

**Keywords:** praise, gesture, motivation, attribution, development

## Abstract

The type of praise children receive influences whether children choose to persist after failure. One mechanism through which praise affects motivation is through the causal attributions inferred from language. For example, telling a child “You got an A on the test because you’re smart,” provides an explicit link between possessing a trait and an outcome, specifically that intelligence causes success. Nonetheless, most praise given to children is ambiguous, or lacks explicit attributions (e.g., “yea” or a thumbs up). To investigate the effects of ambiguous praise on motivation, we randomly assigned 95 5–6-year-old children to a praise condition (verbal trait; verbal effort; verbal ambiguous; or gestural) and measured motivation using task persistence, self-evaluations, and eye fixations on errors. Ambiguous praise, similar to verbal effort praise, produced higher persistence and self-evaluations, and fewer fixations on error after failure compared to verbal trait praise. Interestingly, gestures produced the highest self-evaluations. Thus, praise without explicit attributions motivated as well or better than praise explicitly focused on effort, which may suggest that children interpret ambiguous praise in the most beneficial manner.

## INTRODUCTION

A player scores a goal in a soccer match and her coach gives her a high five. A teacher says, “Awesome!” after a student solves a difficult math problem. After seeing a child’s perfect score on a spelling test, a parent says, “You are so smart!” In each case, the adult has given praise to a child; however, the praise above differs in three ways. First, the praise differs in the modality in which it was conveyed—verbal or gestural. Second, some types of praise provide explicit attributions, or links between causes and outcomes. For example, saying, “You got an A, you are so smart!” explicitly links intelligence to the outcome whereas saying, “Awesome” is ambiguous because it doesnot provide an explicit attributional link. Third, some types of praise explicitly link outcomes to stable, unchangeable traits. For example, praise such as “You are smart” links outcomes to stable traits like intelligence. Other types of praise (e.g., “You worked hard”) explicitly link outcomes to malleable factors such as effort.

Previous research on praise and motivation has largely focused on verbal praise that provides explicit causal links with outcomes. This research has demonstrated that children who receive verbal praise directed toward stable, unchangeable traits (“You’re a good drawer”) tend to show lower motivation (i.e., persistence and self-evaluations) after failure than those who receive praise directed explicitly toward effort (“You did a good job drawing”; Kamins and Dweck, 1999; Cimpian et al., 2007; Zentall and Morris, 2010). However, as illustrated in the examples above, these two types of praise only represent a subset of the praise children receive. The majority of praise children receive is *ambiguous* (e.g., “yea”), which provides no explicit attributions. Gunderson et al. (2013) reported that verbal ambiguous praise (e.g., Wow!) accounted for 66% of praise parents gave to their toddlers during unstructured play. Ambiguous praise can also be conveyed through gestures, such as thumbs up or a high five. Given its ubiquity in natural language, it is surprising that we could find no experimental research to date investigating what effect, if any, ambiguous praise has on motivation. The present study investigated the effect of verbal ambiguous and gestural praise on young children’s motivation.

Changing a child’s explanations for success and failure (i.e., *attributions*) is a likely mechanism through which praise influences motivation (Mueller and Dweck, 1998; Weiner, 2011). Verbal trait praise explicitly links stable, uncontrollable traits (e.g., intelligence) to success/failure, while verbal effort praise explicitly links controllable factors (e.g., hard work) to success/failure. For example, when a child is told, “You’re a good drawer” after drawing a picture, this praise provides an explicit attributional framework in which the trait of being a good drawer is linked with the outcome. Subsequently, errors threaten the possession of this trait as demonstrated by increased visual attention (i.e., fixations) to errors (Zentall and Morris, 2012). Children who receive verbal trait praise and then experience errors in their work tend to demonstrate decreased task motivation (Cimpian et al., 2007; Zentall and Morris, 2012). Although verbal effort praise has a more positive impact on motivation, attributions from verbal trait praise are more heavily weighted than attributions conveyed through verbal effort praise (Zentall and Morris, 2010). Specifically, when children were given different proportions of verbal trait and verbal effort praise, they persisted only when 75% of the praise was directed toward effort. These results suggest that motivation can be undermined by even a small amount of verbal trait praise. This attributional model will be used to examine the relation between praise and motivation (e.g., Barker and Graham, 1987; Cimpian et al., 2007; Gunderson et al., 2013; Brummelman et al., 2014), which explains why children demonstrate lower motivation after receiving verbal trait praise compared to verbal effort praise (see Henderlong and Lepper, 2002 for a review of alternative models).

Because nearly all research to date has focused on explicit praise types, little is known about how ambiguous praise, either verbal or gestural, will influence motivation. Returning to the example above, a teacher saying “Awesome” to a student would be an example of verbal ambiguous praise. Ambiguous praise provides no explicit attribution of the cause of success/failure, thus if explicit attributions are necessary, ambiguous praise should have no effect on motivation. Another possibility is that ambiguous praise provides implicit attributions. For example, gestures such as a “thumbs up” are *emblems—*widely used gestures with a shared meaning (Goldin-Meadow, 2011). If a “thumbs up” has a shared meaning of “good job,” this may suggest a link between an outcome and a child’s effort. Following this example, the shared meaning of the emblem (or a verbal type of ambiguous praise) should predict its effect on motivation. Specifically, if ambiguous praise is interpreted as effort praise, then it should increase motivation; if ambiguous praise is interpreted as trait praise, then it should reduce motivation.

The present study investigated the effects of explicit and ambiguous verbal or gestural praise on young children’s motivation and visual attention to error. We compared verbal trait and verbal effort praise to verbal ambiguous praise (“Yea”), and two types of gestural praise, one expressed verbally (high five) and the other expressed as a gesture (thumbs up) to account for differences in the way the praise was delivered. Irrespective of the effects of ambiguous praise, verbal trait praise was expected to produce significantly lower levels of motivation than the other conditions, because this type of praise explicitly links stable, uncontrollable traits (e.g., intelligence) to success/failure. If explicit attributions are necessary, children who receive verbal effort praise should have the highest motivation, whereas children who receive verbal trait should have the lowest levels of motivation. Ambiguous praise (verbal or gestural), conveying no explicit attributions, would fall in between.

Alternatively, ambiguous praise may be interpreted as providing an attribution. For example, if ambiguous praise is interpreted as effort praise (e.g., a *thumbs up* is interpreted as meaning “good job”), then motivation outcomes should be similar to those associated with verbal effort praise (i.e., high persistence and self-evaluations scores, low visual fixations on error). If ambiguous praise is interpreted as trait praise, the motivation outcomes should be similar to verbal trait praise (i.e., lower persistence and self-motivation scores and higher visual fixations). Given the lack of previous research on this topic, the impact of ambiguous praise was an exploratory question.

To investigate the effects of praise types on motivation, we modified a task used in several previous experiments (Cimpian et al., 2007; Zentall and Morris, 2010, 2012), in which children hear stories about themselves drawing pictures and receive praise for drawing pictures without errors. Although children do not directly experience successes or failures, this method has produced clear effects of praise type (Cimpian et al., 2007; Zentall and Morris, 2010, 2012), such that children who heard stories about themselves receiving verbal trait praise were less motivated than those who received verbal effort praise. Moreover, those findings were similar to experiments in which the children experienced actual successes and failures (e.g., Mueller and Dweck, 1998). Motivation is measured using a series of questions that require explicit, verbal responses about task persistence (e.g., Would you like to draw this again or try something else?) and self-evaluation (e.g., Does this make you feel happy or sad?) based on those used in previous studies of motivation (e.g., Cimpian et al., 2007).

Although verbal responses were used in multiple experiments detecting differences among children’s motivation, young children have limited abilities to provide verbal explanations for their reasoning (Woolley, 2006). Therefore, in addition to verbal explanations, we measured visual attention to error through eye tracking as an objective, behavioral measure. Increased visual fixations on error are related to decreases in persistence, demonstrating its usefulness as a converging measure of motivation (Zentall and Morris, 2012). We used verbal trait and verbal effort praise as de-facto control conditions because their effects have been consistently reported in the literature (Mueller and Dweck, 1998; Kamins and Dweck, 1999; Cimpian et al., 2007; Zentall and Morris, 2010, 2012). In addition to hearing stories about their own errors, children were shown pictures “drawn by other children,” some with and some without errors, as a pretest and post-test. The pretest and post-test was included to measure attention to errors (i.e., missing features) in general before and after receiving the praise. Before receiving any type of praise, the conditions should be roughly equal in the attention to missing features, which allows us to compare differences at post-test to measure the effect of praise between conditions. Because the pictures with the missing features are no longer novel on the second presentation, visual attention should decrease. However, Zentall and Morris (2012) found that children who received verbal trait praise increased the number of error fixations from pretest to post-test. Alternatively, children who received verbal effort praise showed a decrease in visual fixations from pretest to post-test, suggesting that errors became more salient for children who received verbal trait praise, even when the pictures were made by “another child.” The current study investigated how children who received ambiguous praise responded to errors compared to those who received verbal effort or verbal trait praise.

## MATERIALS AND METHODS

### PARTICIPANTS

Ninety-five kindergarteners (*M* = 5.73, SD = 0.22; 75% male, 86% white) were recruited from a public school in the Midwestern U.S. Children were randomly assigned to one of five conditions (Verbal trait, Verbal effort, Verbal ambiguous, and two types of gestural ambiguous praise: thumbs up and high five).

### PROCEDURE

First, children were shown four pretest pictures “drawn by another child.” The experimental phase followed in which children heard stories about drawing successful pictures for their teacher and receiving praise. Children were shown four drawings that they were asked to pretend they had drawn themselves. After the four “successful” scenarios, children were asked four self-evaluation questions. Following these questions, children were given two more drawing scenarios in which they were told to pretend that they did not successfully complete the pictures (i.e., “failure” scenarios). After the “failure” scenarios, persistence, self-evaluations, and eye fixations on errors were measured. Finally, children were shown the four pretest pictures once more as a post-test. We will briefly describe each part of the procedure below; however, the entire script including coding guidelines are provided on our website.

The procedure was adapted from Cimpian et al. (2007) and Zentall and Morris (2012). Children participated in a quiet room away from their classrooms. Each child was seated in front of a Tobii T-60 eye tracker monitor and the eye tracker was calibrated with a 9-point calibration. All pictures described to the children were shown on the eye-tracking monitor. After participating, children received a small toy. The University Institutional Research Board approved our procedures and data collection followed SRCD ethical standards for research with children.

#### Pretest and post-test

Children were shown four pictures, two with missing features (e.g., elephant without a trunk), and were told, “These are pictures other children drew. Some have missing parts.” This framing was used so that a child would not interpret these errors as her own, but as “other children’s errors.” Children were shown these pictures but were not asked to evaluate them as in the Experimental phase. As mentioned above, the purpose of the pretest was to establish a baseline for the number of fixations children produced related to missing parts (i.e., errors) and to better compare the number of fixations after receiving different types of praise controlling for the influence of missing features.

#### Experimental phase

Each child was told that they would be playing a pretending game in which the child would hear stories about a teacher named “Debbie” who will ask them to draw a series of pictures. The child was also told that they would see the picture they “drew” on the computer screen. Each child heard four stories about drawing pictures [e.g., “One day you were playing at the drawing table. Teacher Debbie said, “(*Child’s name)*, will you make a cow for me?” and you said “OK, teacher.” When Teacher Debbie came over and saw the cow you drew she said, “That looks like a cow”]. After hearing this “successful” scenario, the child then received one type of praise related to the drawing [*verbal trait*: “You are a good drawer,” *verbal effort*: “You did a good job drawing,” *verbal ambiguous*: “Yea,” and *gestural ambiguous*: thumbs up: “Your teacher does this (*experimenter demonstrated thumbs up)*,” or high five “Your teacher gives you a high five”]. After the praise, children were shown a corresponding picture on the eye tracker screen. These experimental pictures were different from those shown in the pretest/post-test. It is important to note that children never drew pictures. Children were then asked four self-evaluation questions, followed by two “failure” trials about drawing pictures with errors. For the “failure” trials (hereafter “own” errors), children were told two stories about drawing pictures containing errors (e.g., a cat without ears), followed by corresponding pictures. Then, children were asked the same four self-evaluation questions and four persistence questions. Following the post-test, each child heard stories about successfully fixing their “own” errors and were praised, “You found a really good way to draw the …”

### MEASURES

#### Persistence

Following Cimpian et al. (2007) and Zentall and Morris (2010, 2012), after the “failure” scenario, each child was asked four persistence questions (e.g., “If you had a chance to do something tomorrow, would you draw or do something else?”). Each response was coded as to whether it demonstrated persistence (e.g., draw again = 1, do something else = 0). There were two open-ended persistence questions: one for each “failure” scenario (e.g., “Think about the story where you drew a cat and forgot the ears. What would you do now?”). The authors coded these responses independently with Cohen’s kappa = 0.96 before discussion. The four persistence scores were averaged to create a persistence composite score.

#### Self-evaluations

Following Cimpian et al. (2007) and Zentall and Morris (2010, 2012), children were asked four self-evaluation questions (e.g., “Did what happened in the dog story make you feel like you were good at drawing or not good at drawing?”; “Do you like the dog that you drew or do you not like it?”). Each response was coded as to whether it demonstrated a positive self-evaluation (e.g., good at drawing = 1, not good at drawing = 0). Two self-evaluation composite scores were calculated by averaging over the four questions for the “successful” scenarios (*pre-failure self-evaluation*) and “failure” scenarios (*post-failure self-evaluation*).

#### Visual attention to error

Areas of interest (AOI) were defined around missing features (e.g., the cat’s missing ear) for each picture. The number of fixations (hereon fixation count) and fixation durations within each AOI were recorded on pretest and post-test pictures (framed as “other children’s errors), and for pictures of their “own” errors. Fixations were of primary interest following Zentall and Morris (2012) who found that increases in the number of fixations were related to lower persistence. Fixations allow for *process tracing,* an objective measure of the path of attention that is closely related to task goals (Hayhoe and Ballard, 2005).

#### Interpretations of ambiguous praise

Finally, a subsample covering all conditions (*n* = 32) was asked to respond an open-ended question to define the three types of ambiguous praise used in this study. Specifically, “What does it mean if the teacher gives a high five/gives a thumbs up/says “Yea”?” These responses were recorded and tallied for each type of praise.

## RESULTS

A series of one-way ANOVAs were used to compare the four praise conditions on self-evaluation (pre- and post-“failure”), persistence, and the mean number of visual fixations on errors. Children who received verbal trait praise had significantly lower persistence scores, *F*(4,94) = 20.15, *p* = 0.0001, η^2^ = 0.473 than did children in all other conditions (see **Table 1**). Pre-“failure” self-evaluation scores did not differ significantly across praise conditions, *F* (4,94) = 1.007, *p* = 0.408 but post-“failure” self-evaluation scores differed significantly, *F*(4,94) = 25.307, *p* = 0.0001, η^2^ = 0.529. *Post hoc* tests demonstrated that children who received verbal trait praise had significantly lower self-evaluation scores than all other conditions, and both gestural conditions had significantly higher self-evaluation scores than did all other conditions (see **Table 1**).

**Table 1:** Mean persistence, self-evaluation, and fixation scores by condition (standard deviations in parentheses).

	Verbal trait	Verbal effort	Verbal ambiguous	Gesture: thumbs up	Gesture: high five
N = 95	N = 20	N = 20	N = 19	N = 17	N = 19
Persistence	0.43_a_ (0.27)	0.85_b_ (0.22)	0.87_b_ (0.23)	0.91_b_ (0.20)	0.92_b_ (0.13)
Post-“Failure” Self-Evaluation	0.28_a_ (0.24)	0.73_c_ (0.28)	0.86_c_ (0.24)	0.91_b_ (0.19)	0.96_b_ (0.12)
Fixations on “Own” errors	10.50_a_ (4.45)	6.70_b_ (3.23)	6.11_b_ (3.13)	5.35_b_ (2.69)	7.05_b_ (2.63)
Fixations on “Other Children’s” Errors (post-test)	13.35_a_ (3.99)	5.50_b_ (2.61)	5.58_b_ (3.91)	5.82_b_ (2.94)	4.74_b_ (2.26)
Mean fixation duration on own errors in Milliseconds	373 (0.85)	364 (0.72)	390 (0.62)	359 (0.80)	388 (0.55)

*Means with different subscripts are significantly different at the p < 0.05 level based on LSD post hoc tests.*

Visual attention to error was also compared across conditions. Children who received verbal trait praise produced significantly more fixations on their “own” errors, *F*(4,94) = 6.896, *p* = 0.0001, η^2^ = 0.235 than did children in all other conditions (see **Table 1**). No significant differences were found among the other conditions. For “other children’s” errors, there were no differences in pretest fixation counts across praise conditions, *F*(4,94) = 1.46 *p* = 0.37; however, at post-test, children in the verbal trait praise condition produced significantly more fixations on errors than did any other condition, *F* (4,94) = 25.3, *p* = 0.0001, η^2^ = 0.519. We analyzed the average duration of fixations on missing features by praise condition to investigate the possibility that some children may have produced fewer fixations because they were spending more time looking at errors. The average fixation duration did not differ across conditions, *F*(4,94) = 1.6, *p* = 0.32 (see **Table 1**), demonstrating that fixation counts were an accurate index of attention to error.

Responses from the subsample of children who were asked to define each type of ambiguous praise (verbal ambiguous, gestural- thumbs up, and gestural-high five) were surprisingly consistent. *Thumbs up* was described as meaning “good job” (68%) or “good” (23%), *high five* was described as meaning “good job” (65%) or “good” (23%), and “*Yea”* was described as meaning “the teacher was happy” (42%), “good job” (26%), and “good” (23%). None of the children’s responses for the three types of ambiguous praise were related to traits.

## DISCUSSION

This study is the first to demonstrate that ambiguous verbal and gestural praise, i.e., praise without explicit attributions, positively influence motivation. We discuss two novel findings. First, explicit attributions appear to be unnecessary for praise to influence motivation positively. Praise without explicit attributions explaining the causes of success and failure (e.g., yea!) was as motivating as praise with explicit attributions (“You did a good job drawing”), more specifically, the verbal and gestural praise used in our study produced positive motivational effects similar to verbal effort praise. This is notable because, although frequently used by parents and teachers, no previous research had investigated their effects on motivation. Second, gestural praise, although similar to explicit verbal effort praise on children’s persistence, produced unique benefits for positive self-evaluations. Children receiving gestural praise evaluated themselves and their drawings more favorably than children receiving all types of verbal praise.

Recall that attributions influence motivation by providing information about the degree to which performance is stable and under a child’s control (Henderlong and Lepper, 2002). Specifically, verbal effort praise explicitly links successes and failure to factors under a child’s control (e.g., effort), whereas, verbal trait explicitly links successes and failure to stable factors that are not in a child’s control (e.g., traits; Mueller and Dweck, 1998; Kamins and Dweck, 1999; Henderlong and Lepper, 2002; Cimpian et al., 2007; Zentall and Morris, 2010, 2012; Dweck, 2011). In turn, reactions to errors (i.e., failure) differ based on their attributions. For example, when a child attributes success to traits, such as intelligence, and then experiences failure, s/he is less likely to persist because failure threatens the belief that s/he is intelligent (Blackwell et al., 2007; Dweck, 2011).

Although previous research has investigated praise that provides explicit links between effort or traits and performance (e.g., *You got an “A” on the test. You are smart!”*), the majority of the praise children receive is ambiguous (i.e., lacks explicit attributions). Thus, the present study tested whether explicit attributions were necessary to influence motivation. Our results indicate that motivation was positively influenced by praise without explicit attributions. Consistent with previous findings (e.g., Kamins and Dweck, 1999), children who received explicit attributions in the form of verbal trait praise had the lowest scores on motivation. These findings offer further support that verbal trait praise makes clear and stable attributional links to the unchangeable trait (Cimpian et al., 2007); in this case drawing ability was suggested as the causal factor influencing the quality of a child’s drawings. In turn, error or failure, poses a threat to possessing that trait by providing evidence to the contrary. Children, in turn, decreased task persistence and enjoyment.

Ambiguous praise appears to provide implicit attributions similar to that of verbal effort praise. The evidence from this experiment provides support for this in two ways. First, children given verbal ambiguous or gestural praise tended to persist after errors, had positive evaluations of their work, and did not fixate on their errors as much as children given verbal trait praise. Second, children defined ambiguous praise as a way of positively evaluating performance (e.g., “good job”). Importantly, none of the children described verbal ambiguous or gestural praise as being related to traits (e.g., “I am a good drawer”).

These results suggest that effective praise highlights factors within a child’s control, specifically effort, regardless of whether the attributional links are explicit. Our results demonstrate that children who received explicit verbal praise for effort or ambiguous praise interpreted it as effort praise, which positively influenced their motivation. These data suggest that there are benefits to children linking praise to effort, even if the attribution is implicit, compared to trait praise, which highlights factors that are not under their control, such as ability.

One of the most interesting and surprising findings was that children who received gestural praise felt more positive about themselves and their drawings than those receiving all other types of praise. This result suggests that although children defined the gestures using language associated with positive performance evaluations, these gestures appear to have additional meaning, which increases young children’s positive feelings about their work and themselves. It is not clear why gestural praise had this unique effect on self-evaluations. More research into how gestures and verbal forms of praise are used in naturalistic settings is needed. For example, if adults typically reserve high fives for exceptional work, rather than average work, children may associate high fives with exceptional work, which could account for elevated self-evaluations.

In conclusion, ambiguous praise, i.e., praise without explicit attributions, can be used to motivate young children effectively. Verbal ambiguous praise (“*Yea”*) was as motivating as verbal praise for effort (“*You did a good job drawing”*). Moreover, gestural praise (thumbs up or high five) may be the optimal form of praise—as demonstrated by children’s increased persistence and positive feelings about themselves and their work.

## Conflict of Interest Statement

The authors declare that the research was conducted in the absence of any commercial or financial relationships that could be construed as a potential conflict of interest.
