# Supplementary material for: High fives motivate: the effects of gestural and ambiguous verbal praise on motivation
Source: Front Psychol. 2014 Aug 27;5:928. doi: 10.3389/fpsyg.2014.00928 (PMC4145712; doi:10.3389/fpsyg.2014.00928)
Supplement: Supplementary file 1 [file Data_Sheet_1.PDF]

Praise Gesture Script

Pretest Phase

First, I just want to show you some pictures that other kids have drawn. Some of these pictures have missing parts.
(Each picture is shown for five seconds) This is a picture of a bunny. This is a picture of a semi truck. This is a picture of a pick-up truck. And this is a picture of an elephant.

Experimental Phase

Okay, so now we are going to play a pretending game where we will pretend that you will draw some pictures. I am going to tell you a few stories about you and a teacher, Teacher Debbie. Teacher Debbie asks you to draw a picture and then you'll be able to see a picture on this computer. We will pretend that it is the picture that you drew.

1) One day you were playing at the drawing table. Teacher Debbie said, "(Child's name), will you make a cow for me?" and you said "OK, teacher." When Teacher Debbie came over and saw the cow you drew she said, "That looks like a cow." **[Based on the assigned condition, use one of the following types of praise consistently throughout the procedure]** ***Verbal Trait:*** "***You are a good drawer"/ Verbal Effort: "You did a good job drawing"/ Verbal Ambiguous: "Yea"!/ Gestural, Thumbs-up: "and then does this [experimenter gestures a thumbs-up]"/ Gestural, High-five: "and then gives you a high-five"***.
Let's pretend this is the picture that you drew **(shows picture on screen for five seconds).**

2) Another day you were playing at the drawing table and Teacher Debbie said, "(Child's name), will you make a dog for me?" and you said "OK, teacher." When Teacher Debbie came over and saw the dog you drew she said, "That looks like a dog." (same praise as #1). Let's pretend this is the picture that you drew **(shows picture on screen for five seconds).**

3) Pretend one day you were playing at the drawing table and Teacher Debbie said, "(Child's name), will you make a car for me?" and you said "OK, teacher." When Teacher Debbie came over and saw the car you drew she said, "That looks like a car." (same praise as #1).
Let's pretend this is the picture that you drew **(shows picture on screen for five seconds).**

4) One day you were playing at the drawing table and Teacher Debbie said, "(Child's name), will you make a sheep for me?" and you said "OK, teacher." When Teacher Debbie came over and saw the sheep you drew she said, "That looks like a sheep. (same praise as #1). Let's pretend this is the picture that you drew **(shows picture on screen for five seconds).**

Self-evaluation questions (from Cimpian et al., 2007)
Before we go on I'm going to ask you a few questions about the **sheep** story.
(a) Do you like the sheep that you drew or do you not like it?

- How much do you like/not like it? Do you REALLY LIKE IT? Do you SORT OF LIKE IT? Do you SORT OF NOT LIKE IT? Or do you REALLY NOT LIKE IT?

(b) Did what happened in the sheep story make you feel happy or sad?

- How happy/sad? Does it make you feel REALLY HAPPY? Does it make you feel SORT OF HAPPY? Does it make you feel SORT OF SAD? Or Does it make you feel REALLY SAD?

(c) Did everything that happened in the sheep story make you feel like you were good at drawing or not good at drawing?
(d) Did everything that happened in the sheep story make you feel like you were a good boy/girl or not a good boy/girl?

The coding of Self-evaluation questions followed Cimpian et al. (2007)

(a) REALLY LIKE IT? = 1
SORT OF LIKE IT? = 1
SORT OF NOT LIKE IT? = 0
REALLY NOT LIKE IT? = 0
(b) REALLY HAPPY? = 1
SORT OF HAPPY? = 1
SORT OF SAD? = 0
REALLY SAD? = 0
(c) Good at drawing = 1
Not good at drawing = 0
(d) good boy/girl = 1
not a good boy/girl = 0

5) Another day you were playing at the drawing table. After a little while, Teacher Debbie says, "(Child’s name), will you make a cat for me?" and you say "OK, teacher." You look at what you did and think to yourself, "Uh-oh, the cat doesn’t have any ears," but you want to show the teacher the cat you drew and so you say "Teacher, I drew a cat for you." The teacher looks at the cat you drew and says, “That doesn’t look like a cat; it has no ears.”
Let’s pretend this is the picture that you drew **(shows picture on screen for five seconds).**

Let’s see what happens in the next story.
6) Another day you were playing at the drawing table. After a little while, Teacher Debbie says, "(Child's name), will you make a bus for me?" and you say "OK, teacher." You look at what you did and think to yourself, "Uh-oh, the bus doesn't have any wheels," but you want to show the teacher the bus you drew and so you say, "Teacher, I drew a bus for you." The teacher looks at the bus you drew and says, "That doesn't look like a bus; it has no wheels."
Let’s pretend this is the picture that you drew **(shows picture on screen for five seconds).**

Self-evaluation questions
Okay, now I’m going to ask you some questions about this **bus** story.
(a) Do you like the bus that you drew or do you not like it?

- How much do you like/not like it? Do you REALLY LIKE IT? Do you SORT OF LIKE IT? Do you SORT OF NOT LIKE IT? Or do you REALLY NOT LIKE IT?

(b) Did what happened in the bus story where you forgot the ears make you feel happy or sad?

- How happy/sad? Does it make you feel REALLY HAPPY? Does it make you feel SORT OF HAPPY? Does it make you feel SORT OF SAD? Or Does it make you feel REALLY SAD?

(c) Did everything that happened in the bus story where you forgot the wheels make you feel like you were good at drawing or not good at drawing?

(d) Did everything that happened in the bus story where you forgot the ears make you feel like you were a good boy/girl or not a good boy/girl?

Persistence questions (from Cimpian et al., 2007)
(e) On another day, if you had a chance to draw one of these again, would you want to draw the bus, the cat, or the sheep? **[shows slide with all three pictures]**
(f) If you had a chance to choose an activity tomorrow, would you draw or would you do something else?
(g) Think about the story where you drew a cat and forgot the ears. What would you do now?
(h) Think about the story where you drew a bus and forgot the wheels. What would you do now?

Persistence question coding follows Cimpian et al. (2007)
(e) Bus = 1 (demonstrates persistence because child failed to draw correctly)
Cat = 1 (demonstrates persistence because child failed to draw correctly)
Sheep = 0 (demonstrates lack of persistence because child already drew it correctly)
(f) Draw = 1(demonstrates persistence)
Something else = 0
(g) Response that indicates persistence (e.g., draw the ears)= 1
Response that does not indicate persistence (e.g., play a puzzle)= 0
(h) Response that indicates persistence (e.g., draw the ears)= 1
Response that does not indicate persistence (e.g., play a puzzle)= 0

Posttest Phase

Now I want to show you these pictures that other kids drew one more time. Remember, some of these pictures have missing parts.
(Each picture is shown for five seconds, these are the same pictures used in the pre-test). This is a picture of a bunny. This is a picture of a semi truck. This is a picture of a pick-up truck. And this is a picture of an elephant.

Okay, so now let’s go back to our pretending game and let’s try the bus again:
7) Another day you were playing and Teacher Debbie says, "Will you make a cat for me?" and you say "OK, teacher." So you work really hard and try to draw a good cat for the teacher. The teacher looks at the cat you drew and says, “You found a really good way to draw the cat. I see it has ears.” Let’s pretend this is the picture that you drew.

Let’s see about the cat:
8) Another day you were playing at the drawing table and Teacher Debbie asks you to make a bus and you say "OK, teacher." So you work really hard and try to draw a good bus for the teacher. The teacher looks at the bus you drew and says, “You found a really good way to draw the bus. I see that it has wheels.” Let’s pretend this is the picture that you drew.

Great! Thanks for playing the game. Would you like to pick a prize for playing?
